# BRWLDA: bi-random walks for predicting lncRNA-disease associations

**DOI:** 10.18632/oncotarget.19588

**Published:** 2017-07-26

**Authors:** Guoxian Yu, Guangyuan Fu, Chang Lu, Yazhou Ren, Jun Wang

**Affiliations:** ^1^ College of Computer and Information Sciences, Southwest University, Chongqing, China; ^2^ Big Data Research Center, School of Computer Science and Engineering, University of Electronic Science and Technology of China, Chengdu, China

**Keywords:** lncRNAs, diseases, lncRNA-disease associations, bi-relational network, bi-random walk

## Abstract

Increasing efforts have been done to figure out the association between lncRNAs and complex diseases. Many computational models construct various lncRNA similarity networks, disease similarity networks, along with known lncRNA-disease associations to infer novel associations. However, most of them neglect the structural difference between lncRNAs network and diseases network, hierarchical relationships between diseases and pattern of newly discovered associations. In this study, we developed a model that performs *B*i-*R*andom *W*alks to predict novel *L*ncRNA-*D*isease *A*ssociations (BRWLDA in short). This model utilizes multiple heterogeneous data to construct the lncRNA functional similarity network, and Disease Ontology to construct a disease network. It then constructs a directed bi-relational network based on these two networks and available lncRNAs-disease associations. Next, it applies bi-random walks on the network to predict potential associations. BRWLDA achieves reliable and better performance than other comparing methods not only on experiment verified associations, but also on the simulated experiments with masked associations. Case studies further demonstrate the feasibility of BRWLDA in identifying new lncRNA-disease associations.

## INTRODUCTION

The central dogma of molecular biology assumes genetic information is stored in protein-coding genes. There are about 20000 human protein-coding genes, accounting for less than 2% of the human genome, and more than 98% of the genome does not encode proteins [1–5] but produces tens of thousands of non-coding RNAs (ncRNAs). RNAs are intermediary molecules between a DNA sequence and its encoded proteins [6]. Among heterogeneous subtypes of ncRNAs, long ncRNAs (lncRNAs), are similar to mRNAs in gene structure, with length greater than 200nt [7]. Rapid influx of biological and medical evidences show that lncRNAs play fundamental and critical roles in various biological processes, such as cell proliferation, differentiation, chromatin remodeling, epigenetic regulation, genomic splicing, transcription, translation [7–12]. Particularly, more and more literature reports that the alterations and dysregulations of lncRNAs are associated with the development and progression of various complex diseases. For example, lncRNA HOTAIR (HOX antisense intergenic RNA) has 100 to approximately 2000-fold expression levels in breast cancer metastases based on quantitative PCR. It controls the pattern of histone modifications and regulates gene expression by binding to histone modifiers, PRC2 and LSD1 complexes [13, 14]. HOTAIR is viewed as a potential biomarker in various types of cancers. By down-regulating H19, an lncRNA confirmed more than 20 years ago [15], the breast and lung cancer cell clonogenicity and anchorage-independent growth can be significantly decreased [16]. In fact, H19 is involved with various diseases and can be used as a potential prognostic biomarker for the early recurrence of bladder cancer [17].

From these concrete associations between lncRNAs and diseases, we can find that it is necessary and promising to collect lncRNA-disease associations as many as possible. However, although experimentally confirmed lncRNA-disease associations have been increasing, the number is still rather small, compared with a large number of lncRNAs and diseases [18–21]. Furthermore, determining the associations between lncRNAs and diseases by wet-lab experiments is very expensive and time consuming. Given that, accurately identifying lncRNA-disease associations by computational models can not only benefit the further biological experiments by saving cost and time, but also assist disease biomarkers detection for disease diagnosis, treatment, prognosis and prevention. In addition, these correctly identified associations can speed up our pace on understanding life process at RNA level.

Some data mining based models have been applied to predict lncRNA-disease associations in large scale. Chen et al. [22] assumed that functionally similar lncRNAs tend to interact with similar diseases, and introduced a Laplacian Regularized Least Squares for LncRNA-Disease Association (LRLSLDA) method to infer novel human lncRNA-disease associations based on lncRNA expression profiles. LRLSLDA computes Gaussian interaction profile kernel similarity for both disease and lncRNAs from known lncRNA-disease associations and lncRNA expression profiles, and then applies the framework of Laplacian Regularized Least Squares to identify potential associations. However, LRLSLDA is suffered from choosing suitable parameters and effectively combining two classifiers. Based on the assumption that functionally related lncRNAs are likely to be associated with phenotypically similar diseases, Sun et al. [23] proposed a global network-based method called RWRlncD to predict lncRNA-disease associations. RWRlncD firstly constructs the lncRNA functional similarity network, disease similarity network and lncRNA-disease association network, and then performs random walk with restart [24] on lncRNA functional network to infer potential associations between lncRNAs and diseases. RWRlncD only takes into account lncRNAs already associated with diseases. For an lncRNA currently not associated with any disease, its association with candidate diseases cannot be inferred. Yang et al. [25] investigated lncRNA-disease associations from a network view to study the connections between lncRNAs and complex diseases. Particularly, based on the fact that both protein coding genes and lncRNAs are associated with human diseases, they constructed a coding-non-coding gene-disease bipartite network, composed with an lncRNA-implicated disease network and a disease-associated lncRNA network, from the available lncRNA-disease associations, and then applied a label propagation algorithm to predict novel lncRNA-disease associations. Obviously, their method asks for sufficient information of non-coding genes, protein coding genes interactions and lncRNAs functional annotations, all of them are incomplete and rather difficult to collect.

These aforementioned methods make use of all verified lncRNA-disease associations in network construction. But in the leave-one-out cross-validation (LOOCV), the test association is treated as unknown for prediction. Given that, the validation is biased toward the test association. In practice, although various types of biological data related to lncRNAs have been accumulated and stored in the public databases (i.e., starBase [26], lncRNAdb [27], NERD [28] and NONCODE [29]), lncRNAs reported to be associated with diseases are still rather limited.

Some efforts moved toward predicting novel lncRNA-disease associations without referring to known associations. Liu et al. [30] predicted potential lncRNA-disease association by integrating known gene-disease associations and gene-lncRNA co-expression relationship. But for a disease temporarily with no related genes, their method cannot predict its associated lncRNAs. Li et al. [31] identified potential associations between lncRNAs and vascular disease based on genome location to globally screen relevant lncRNAs. This genomic location based method has restricted application scope, since not all the lncRNAs have neighborhood genes. Even if an lncRNA has neighborhood genes, this lncRNA may be not functionally related with these neighborhood genes. Zhou et al. [32] assumed that lncRNAs sharing significantly enriched interacting miRNAs tend to be involved with similar diseases and have more functionally related gene sets, and introduced a method called RWRHLD. RWRHLD integrates three networks (miRNA-associated lncRNA-lncRNA crosstalk network, disease-disease similarity network and known lncRNA-disease association network) into a heterogeneous network and applies random walk with restart on this heterogeneous network to predict novel lncRNA-disease associations. Chen [33] proposed a model called HGLDA to predict lncRNA-disease associations by using miRNA-disease interactions and lncRNA-miRNA interactions. HGLDA applies hypergeometric distribution test for each lncRNA-disease pair, and takes the pair sharing common interacting miRNAs as a candidate association. Both RWRLDA and HGLDA are independent from the prior lncRNA-disease associations, and achieve reliable predictions. However, they cannot be applied to lncRNAs without any known miRNA interaction partners.

With the rapidly accumulated biological data, it is possible and necessary to integrate multiple sources of biological data to infer lncRNA-disease associations. Each source provides an incomplete view of the complex mechanism between diseases and biological molecules (including lncRNAs, miRNAs and genes), and integrating multiple related data sources helps to reach a more comprehensive view of the mechanism. Given that, integrating multiple biological data has been one of the most important and attractive topics in the lncRNAs-diseases analysis, and also in many other bioinformatics domains [11, 12, 34–42]. Chen et al. [43] proposed a data integration based method called KATZLDA. KATZLDA integrates known lncRNA-disease associations, lncRNA expression profiles, lncRNA functional similarity, disease semantic similarity and Guassian interaction profiles kernel similarity to predict lncRNA-disease associations. Lan et al. [44] integrated lncRNA sequences, lncRNA-disease associations to construct an lncRNA-lncRNA similarity matrix, and combined lncRNA-disease associations, disease associated genes, protein-protein interactions and Gene Ontology [45] to construct a disease-disease similarity matrix, and then used Karcher mean [46] to fuse these two matrices to predict lncRNA-disease associations based on bagging support vector machine. Chen et al. [47] proposed an Improved Random Walk with Restart for LncRNA-Disease Association prediction (IRWRLDA), which takes advantage of lncRNA-miRNA interactions, miRNA-disease associations, disease semantic similarity from disease MeSH descriptors, lincRNA expression profiles and lncRNA-disease associations to predict potential lncRNA-disease associations. Deng et al. [48], applied a flow propagation algorithm on a network consisted of lncRNA-lncRNA network, protein-protein interactions network, disease similarity network and the associations between each pair of them to predict novel lncRNA-disease associations. These methods achieve significantly improved results than previous methods (i.e., LRLSLDA and RWRlncD). Besides, some other methods (i.e., ILNCSIM and FMLNCSIM [49, 50]) targeted at constructing lncRNA-lncRNA functional network by fusing similarities derived from multiple data sources [43, 47, 49, 50], and predicted potential lncRNA-disease associations based on the functional network. These methods also got improved results than using one (or several) of these data sources alone.

All these network-based or data integration based methods, either do not take into account the structural difference between the network of lncRNAs and that of diseases, or ignore the hierarchical relationship between diseases and pattern of newly discovered associations. To address these issues, in this paper, we presented a new method called Bi-Random Walk on directed bi-relational graph to predict novel lncRNA-disease associations (BRWLDA in short). BRWLDA firstly utilizes lncRNA-miRNA interactions, miRNA-disease associations and lncRNA-gene functional associations to define the lncRNA-lncRNA similarity. The first two datasets are obtained from starBase v2.0 [26] and HMDD [51], and the last one is derived from Gene Reference into Function (GeneRIFs) [52]. Next, BRWLDA constructs a directed bi-relational network between lncRNAs and diseases by viewing each lncRNA (or disease) as a node of that network, and initializes edges between lncRNAs based on the lncRNA-lncRNA similarity, directed edges between diseases based on the Disease Ontology (DO) hierarchy [53] and the edges between lncRNAs and diseases based on lncRNA-disease associations collected from GeneRIFs and LncRNADisease [18]. To account for structural difference between the subnetwork composed with lncRNA nodes and that composed with disease nodes, BRWLDA applies a bi-random (asynchronous) walks [54] on the directed bi-relational network to predict novel lncRNA-disease associations. BRWLDA achieves reliable predictions (with AUC=0.7952 and AUC=0.7940) on disease-oriented and lncRNA-oriented LOOCV, and outperforms previously proposed LRLSLDA [22], RWRlncD [23], RWRHLD [32], ILNCSIM [49] and IRWRLDA [48]. In the simulated experiments with masked associations between lncRNAs and diseases, BRWLDA also obtains higher AUC than these state-of-the-art solutions. In addition, by manually literature mining, 19 lncRNAs in top 20 predictions of BRWLDA are confirmed being associated with three cancers (Breast, Colon and Lung) of wide interests. These examples further demonstrate the effectiveness and potential value of BRWLDA in identifying novel lncRNA-disease associations.

## RESULTS

### Experimental protocols

To quantitatively study the performance of the proposed method and that of other related comparing methods, two different orientations of LOOCV are implemented on experimentally verified lncRNA-disease associations collected from GeneRIFs [52] and LncRNADisease [18]. For disease oriented LOOCV, each disease's association with lncRNAs is left out in turn as the test sample for prediction. For lncRNA oriented LOOCV, each lncRNA's association with diseases is left out in turn to be predicted. These test samples are then evaluated with respect to all the diseases or lncRNAs. Particularly, test samples whose predicted ranks above a given threshold are considered as successful predictions, while those with predicted ranks lower than the threshold are considered as unsuccessful predictions. By varying the threshold, true positive rate (TPR, sensitivity) and false positive rate (FPR, 1-specificity) can be obtained. Sensitivity denotes the percentage of predictions ranking higher than the threshold, and specificity describes the percentage of predictions ranking lower than the threshold. Receiver-operating characteristics (ROC) curve can be drawn by plotting TPR versus FPR at different thresholds, and the area under ROC curve (AUC) is calculated to quantify the prediction performance. The larger the AUC value, the better the performance is, and AUC=0.5equals to random guess.

**Figure 1:**
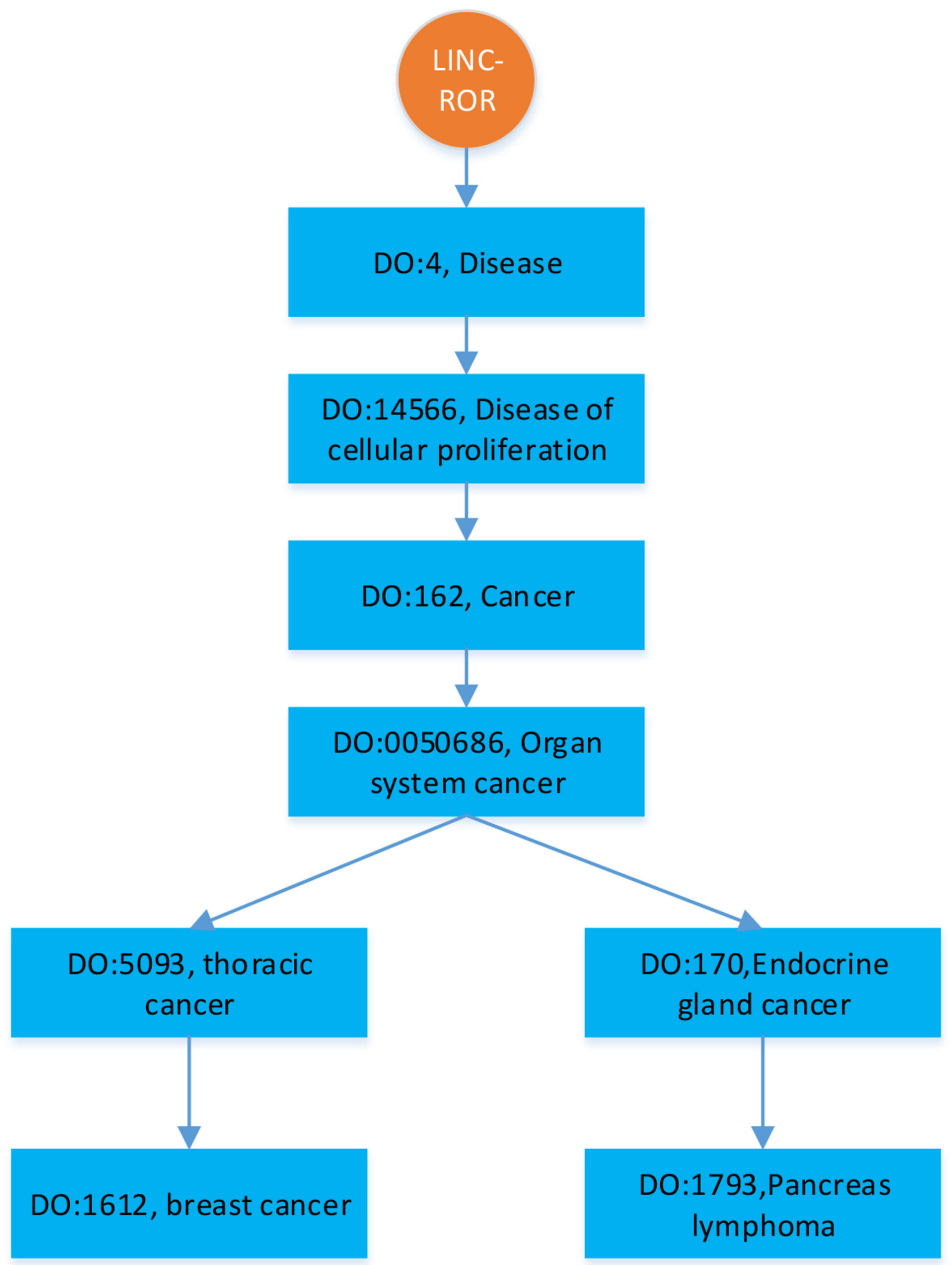
An illustration of hierarchically structured diseases associated with LINC-ROR

To comparatively study the performance of BRWLDA, we compare BRWLDA with other related methods (LRLSLDA [22], RWRlncD [23], RWRHLD [32], ILNCSIM [49] and IRWRLDA [48]) in disease-oriented and lncRNA-oriented LOOCVs, respectively. Next, we simulate the situation of predicting more detailed associations between diseases and lncRNAs. Particularly, if an lncRNA is associated with several diseases (see Figure 1), based on the DO hierarchy, we randomly mask the deepest (specific) diseases (i.e., DO: 1612 and DO: 1793 in Figure 1) in the hierarchy, and then recursively succeed to ancestor diseases (i.e., DO: 5093). This simulated experiment setting is quite different from the widely-used LOOCV, it can assess the ability of BRWLDA in predicting specific diseases (or subtypes of a cancer) associated with lncRNAs. These specific associations may provide more valuable directional clues for complex disease analysis and precise treatments. These clues are also of great interests to medical scientists. In the end, case study with respect to three cancers (Breast, Colon and Lung) of wide interests is performed to validate the effectiveness of BRWLDA.

### Parameter sensitivity analysis

**Figure 2:**
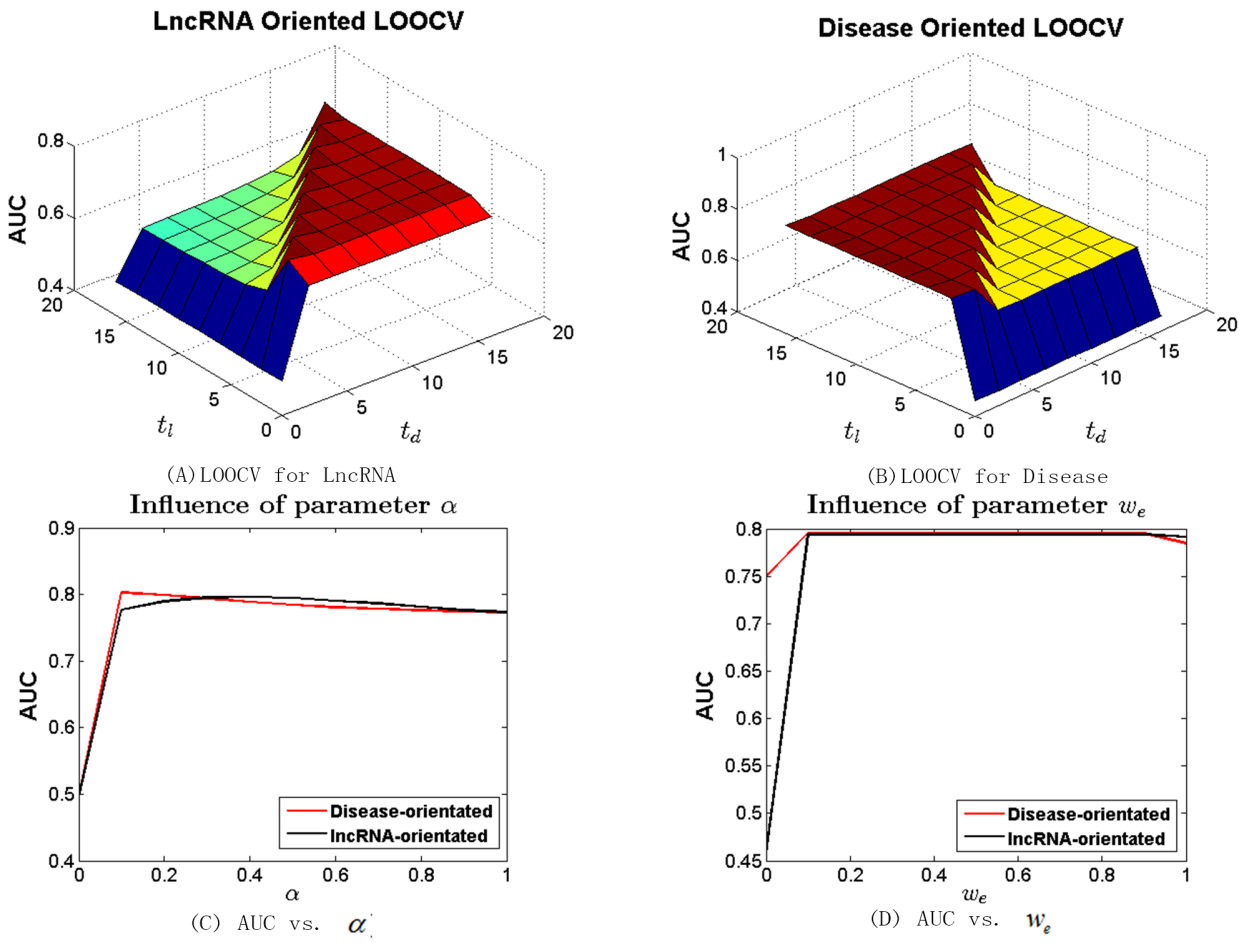
Sensitivity analysis of parameters (*t_l_*, *t_d_* and *α*) of bi-random walks with restart in terms of AUC **(A)** LOOCV for lncRNA **(B)** LOOCV for disease **(C)** AUC vs. α **(D)** AUC vs. *w*e *t_l_* (*t_d_*) control the number of steps for a walk jumping from the lncRNA (disease) subnetwork to disease (lncRNA) subnetwork, *α* controls the restart probability of a random walker. *w_e_* controls the semantic contribution of hierarchically linked disease in the disease similarity computation.

Different from other random walk based approaches [23, 24, 48] that use two parameters (*t* and *α*) to control the steps of global random walks and the restart probability, BRWLDA utilizes two parameters (*t_l_* and *t_d_*) to control the steps of a directed random walker starting from the lncRNA subnetwork and then residing in the disease subnetwork, and the steps of a random walker starting from the disease subnetwork and then stopping in the lncRNA subnetwork. To investigate the influence of these parameters, we increase *t_l_* and *t_d_* from 0 to 16 with step size as 2 in lncRNA (or disease) oriented LOOCV with *α* fixed as 0.3. We vary *α* from 0 to 1 with step size 0.1 and fix both *t_l_* and *t_d_* as 4. The AUC values of BRWLDA under different combinations of *t_l_* and *t_d_* are reported in Figure 2A (lncRNA oriented) and Figure 2B (disease oriented), and the AUC of BRWLDA under different input values of *α* is plotted in Figure 2C.

Obviously, BRWLDA obtains relatively stable AUC when both *t_l_* and *t_d_* are larger than 2. In contrast, its AUC is sharply reduced with *t_l_* = 0 (or *t_d_* = 0),. Another interesting observation is that in the lncRNA oriented LOOCV, BRWLDA gets better AUC when *t_d_* ≥ 2, and in the disease oriented LOOCV, BRWLDA also has good AUC when *t_l_* ≥ 2. That is principally because *t_d_* ≥ 2 (or *t_l_* ≥ 2) enables a walker to move from lncRNA (or disease) nodes to disease (lncRNA) nodes. In this way, potential associations can be predicted. This observation also indicates the necessity of fusing the predicted lncRNA-disease associations started from lncRNAs and those started from diseases. Based on the results in Figure 2A and Figure 2B, in the following experiments, we set *t_l_* = *t_d_* = 4.

In Figure 2C, we can see the AUC with *α* = 0 is the lowest, since no new lncRNA-disease associations can be predicted in this case. When *α* is increasing from 0.4 to 1, AUC of the lncRNA oriented and that of the disease oriented LOOCV both show decreasing trends, but they show different trends when *α* is increasing from 0.1 to 0.4. This fact indicates two random walkers on the directed bi-relational network should be specified with different restart probabilities and also indicates the rationality of applying bi-random walks on the bi-relational network, instead of the global random walks. Given the two oriented LOOCVs have comparable and high AUC around *α* = 0.3, we fix *α* = 0.3 in the following experiments.

We also investigate the parameter *w_e_* in the disease similarity calculation, *w_e_* represents the semantic contribution of links between disease terms in the Disease Ontology. We vary *w_e_* from 0 to 1 with step size 0.1 and reveal the results in Figure 2D. From this figure, we can see that when *w_e_* = 0 both lncRNA and disease-oriented LOOCV have the lowest AUC. That is because diseases are treated as isolated nodes of DO hierarchy and the similarity between diseases are zeros in this extreme setting (*w_e_* = 0). When *w_e_* = 1, the corresponding AUCs are also lower than those when *w_e_* ∈ (0, 1). That is because the semantic contribution between each pair of disease terms differs, a node in a direct acyclic graph (DAG) always gets more contributed information from its directly linked nodes than indirect ones. AUC seems steady when *w_e_* is increasing from 0.1 to 0.9. This trend can be contributed to that the known lncRNA-disease associations are still limited. Based on these observations, to make the semantic contribution differs smoothly, we adopt *w_e_* = 0.9 to measure the similarity between disease terms.

### Results of LOOCV on lncRNA-disease associations

**Figure 3:**
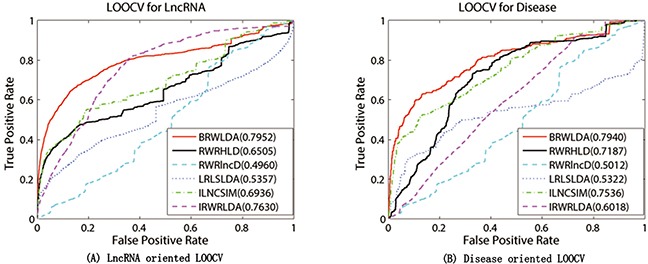
Performance comparison between BRWLDA, RWRHLD, RWRlncD, LRLSLDA, ILNCSIM and IRWRLDA in terms of ROC curve and AUC **(A)** LOOCV for lncRNA **(B)** LOOCV for disease. The left figure is (A) and the right figure is (B) BRWLDA obtains AUCs of 0.7952 and 0.7940 in lncRNA oriented LOOCV and disease oriented LOOCV, respectively. BRWLDA significantly outperforms all the comparing methods and demonstrates its effectiveness in predicting lncRNA-disease associations.

BRWLDA and other comparing methods are applied on the experimentally verified lncRNA-disease associations collected from GeneRIFs [52] and LncRNADisease [18] databases in the framework of LOOCV. Figure 3 plots the ROC curves of these comparing methods and their corresponding AUCs. From this figure, we can clearly see that BRWLDA almost always gets higher true positive rates under the same false negative rates, and achieves higher AUC than these comparing methods. In the lncRNA oriented LOOCV, AUC of BRWLDA is 0.7952, and those of RWRHLD, RWRlncD, LRLSLDA, ILNCSIM and IRWRLDA are 0.6505, 0.4960, 0.5454, 0.6936 and 0.7630, respectively. In the disease oriented LOOCV, AUC of BRWLDA is 0.7940, whereas AUCs of these comparing methods are 0.7188, 0.5012, 5322, 0.7535 and 6018, respectively.

Although RWRHLD utilizes a heterogeneous network similar as the bi-relational network to predict novel associations, it ignores the structural difference between different subnetworks. Given that, RWRHLD is outperformed by BRWLDA. RWRlncD depends on known lncRNA-disease associations to calculate the similarity between lncRNAs. It does not pay attention to the interrelationship between diseases and cannot be applied to lncRNAs (or diseases) without any known lncRNA-disease associations. For this reason, it gets lower AUC than other methods, and even obtains lower AUC than random guess. Both LRLSLDA and ILNCSIM suffer from combining results from two classifiers and selecting the optimal parameter. Furthermore, ILNCSIM overestimates the similarity between lncRNAs, since each left out test association is already utilized to compute the similarity between lncRNAs before LOOCV. ILNCSIM and IRWRLDA generally obtain better ROC curves and larger AUCs than other comparing methods (except BRWLDA). That is because they construct the lncRNA functional network by integrating various data sources, including disease MeSH descriptors, hierarchical structure between disease terms in DO, disease-miRNA associations, lncRNA-miRNA interactions and lncRNA expression profiles. This fact shows that integrating multiple relevant data sources can improve the reliability of lncRNA-disease association prediction.

**Figure 4:**
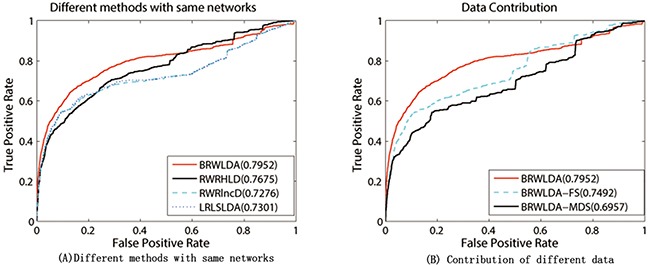
**(A)** Performance of comparing methods under the same input networks. **(B)** Contribution of different data used by BRWLDA. (A) RWRHLD, RWRlncD and LRLSLDA all use the same lncRNA similarity network and disease similarity network constructed by BRWLDA. (B) BRWLDA-FS only uses the functional similarity derived from GO annotations of lncRNAs, BRWLDA-MDS only utilizes the similarity derived from lncRNA-miRNA interactions and miRNA-disease associations, and BRWLDA takes advantage of these two types of similarities.

Since RWRHLD, RWRlncD and LRLSLDA compute the similarity between lncRNAs before lncRNA oriented LOOCV, they are suffered from the overestimation problem in the lncRNA oriented LOOCV. In other words, the left out associations are already used before testing. To reach a comprehensive and fair evaluation of the effectiveness of BRWLDA and of these comparing methods, we use the same lncRNA similarity and disease similarity computed by BRWLDA as the input of these methods, and report the results in Figure 4A. By referring to Figure 3A, we also find that, by fusing multiple data sources, RWRHLD improves the AUC from 0.6505 to 0.7675, RWRlncD increases from 0.496 to 0.7276, and LRLSLDA improves from 0.5454 to 0.7301. These improvements show the potential of data fusion in identifying novel lncRNA-disease associations. In addition, we can also find BRWLDA gets the best ROC curves among these comparing methods and its AUC is larger than the second best RWRHLD. These results again support the advantage of applying bi-random walks on the directed bi-relational network for predicting potential lncRNA-disease associations.

We further investigate the contribution of two components of lncRNAs' similarity. For this investigation, we introduce two variants of BRWLDA: i) BRWLDA-FS only employs functional similarity derived from GO annotations of lncRNAs; and ii) BRWLDA-MDS only utilizes the similarity derived from lncRNA-miRNA interactions and miRNA-disease associations. Figure 4B reports the results of these two variants and BRWLDA. We can observe that BRWLDA generally achieves better performance than these variants. From the results in Figure 4B, we can conclude that integrating GO annotations of lncRNAs and related miRNAs helps to build a more functional-coherent lncRNA-lncRNA network than using single data source alone. The improvement of BRWLDA to BRWLDA-MDS is more prominent than to BRWLDA-FS. That is possible because GO terms are overlapped with DO terms in certain extent and using GO annotations of lncRNAs can more accurately reflect the functional similarity between lncRNAs than miRNA-disease associations and lncRNA-miRNA interactions.

### Result of masked lncRNA-disease associations

**Figure 5:**
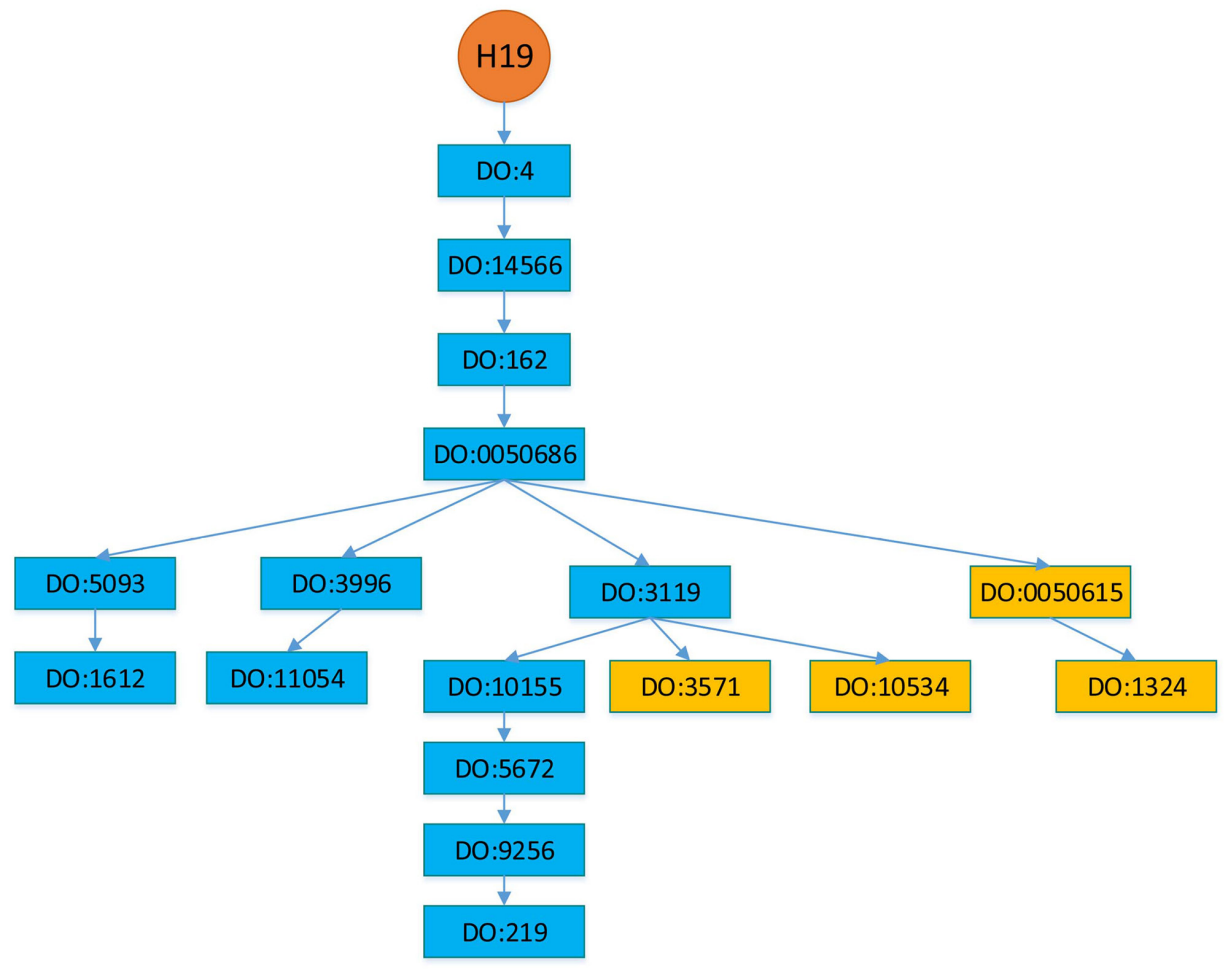
Diseases associated with lncRNA H19 Diseases in the blue rectangles were associated with H19 before 2005, whereas diseases in the orange rectangles are discovered to be associated with H19 after 2005. Particularly, lung cancer (DO:1324) was in 2006, liver cancer (DO:3571) was in 2011 and gastric cancer (DO:10534) was in 2014. The hierarchy is adopted from Disease Ontology.

In this section, we setup a more realistic experimental scenario to investigate the performance of BRWLDA and that of other comparing methods. DO and lncRNA-disease associations are continuously updated, and newly appended associations with an lncRNA often correspond to descendants of the diseases in the DO hierarchy that are already associated with this lncRNA. In Figure 5, we provide an example of H19 about how it was sequentially associated with descendant diseases. In 2005, H19 was reported to be associated with breast cancer (DO:1612), bladder cancer (DO:11054) and colon cancer (DO:219). In 2006, it was reported to be associated with lung cancer (DO:1324), in 2011 it was recognized to be associated with liver cancer (DO:3571), and in 2014 it was reported to be associated with gastric cancer (DO:10534) [16, 55, 56], these newly discovered associations correspond to descendants of the diseases already associated with H19. From the pattern of newly associated diseases of H19, we can find the DO hierarchy should be used to identify new diseases associated with lncRNAs. In other words, these newly appended associations depict more specific complex diseases (or subtypes of a cancer) associated with lncRNAs. Accurately predicting these specific associations is more interesting and helpful for precise diagnosis and treatment.

Motivated by this viewpoint, we assume currently available disease associations of lncRNAs are complete. For a direct acyclic graph (DAG) constructed by a particular lncRNA's associated diseases, we randomly and recursively mask the diseases corresponding to leaf nodes in the DAG of an lncRNA. We regard these masked diseases as newly discovered associations and evaluate the performance of these comparing methods on predicting these associations. In the experiment, we use *k* to denote the number of masked associations of each lncRNA. For example, *k* = 3 indicates 3 diseases associated with an lncRNA are masked. If the number of associated diseases of an lncRNA is less than *k*, we do not mask all the diseases, but keep at least one disease unmasked. To reduce random effect, we repeat each comparing method under each *k* (1, 3, 5) for 10 independent rounds and report the average result and standard deviation in Table 1. Results in **boldface** are significantly superior to other results in the same setting, with statistical significance is checked by pairwise *t*-test at 95% significance level.

**Table 1:** AUC of BRWLDA, RWRHLD, RWRlncD, ILNCSIM and LRLSLDA on predicting masked lncRNA-disease associations

	*k*		
	1	3	5
BRWLDA	**0.9888±0.0004**	**0.9548±0.0003**	**0.9275±0.0005**
RWRHLD	0.9830±0.0010	0.9414±0.0011	0.7801±0.0008
RWRlncD	0.9817±0.0008	0.9444±0.0008	0.9004±0.0025
ILNCSIM	0.9417±0.0009	0.9058±0.0013	0.8528±0.0015
IRWRLDA	0.9405±0.0005	0.9321±0.0019	0.9001±0.0015
LRLSLDA	0.7871±0.0012	0.7619±0.0005	0.7447±0.0027

From Table 1, we can clearly find that BRWLDA outperforms these comparing methods across different input values of *k*. ILNCSIM takes LRLSLDA as its classifier, since it not only utilizes disease Gaussian interaction profile kernel similarity, but also the DO hierarchical structure to measure the similarity between diseases, so it achieves better results than the latter. However, ILNCSIM does not take into account the DO hierarchy in the training and prediction process, it is always outperformed by BRWLDA. For the same reason, IRWRLDA is also outperformed by BRWLDA. Although RWRHLD constructs a heterogeneous network, which is similar as the directed bi-relational network used by BRWLDA, its AUC is comparable with BRWLDA when *k* = 1. However as *k* increasing, the performance margin between BRWLDA and RWRHLD quickly increases. That is because RWRHLD neither takes into account the DO hierarchy, nor the structural difference between lncRNA subnetwork and disease subnetwork. In fact, it applies global random walk with restart on the heterogeneous network. RWRlncD calculates the similarity between lncRNAs by referring to disease structural similarity, and applies random walk with restart only on lncRNA functional similarity network, it initially obtains similar AUC as BRWLDA and latter gets much lower AUC than BRWLDA. The reason is that RWRlncD also neglects the DO hierarchy. These observations again corroborate the advantage of bi-random walks on the directed bi-relational network in predicting lncRNA-disease associations, and also suggest that DO hierarchy should be utilized in predicting potential lncRNA-disease associations.

To further study whether an association prediction method has the ability to predict novel lncRNAs associated with a disease that currently is not associated with any lncRNA. To compare the performance in this regard, we apply BRWLDA, ILNCSIM and RWRHLD for 6 critical cancers, including breast cancer, colon cancer, bladder cancer, lung cancer and stomach cancer. These three methods are adopted since they work better than other comparing methods in disease oriented

LOOCV. For a disease *d*, we remove all its related lncRNAs to simulate the case, and then we apply these methods to predict lncRNAs associated with this disease. Next, we count the number of confirmed lncRNAs in Top *k* ranks for each disease, and *k* equals to the number of removed lncRNAs of this disease.

From Table 2, we can find that BRWLDA outperforms ILNCSIM and RWRHLD in identifying lncRNAs associated with these diseases, whose associated lncRNAs are completely unknown. Although ILNCSIM identifies one more confirmed lncRNA than BRWLDA for liver cancer, the latter always correctly identifies more related lncRNAs of other cancers than the former and RWRHLD. This global observation indicates that BRWLDA can more accurately identify lncRNAs associated with the diseases, whose related lncRNAs are completely unknown.

**Table 2:** The numbers of the confirmed lncRNA-disease associations predicted by BRWLDA, ILNCSIM and RWRHLD under Top *k* rank

Disease name	Number of known associated lncRNAs(*k*)	methods		
		BRWLDA	ILNCSIM	RWRHLD
breast cancer	26	14	12	9
colon cancer	6	4	3	3
bladder cancer	13	9	7	5
liver cancer	31	20	21	13
lung cancer	25	16	16	12
stomach cancer	14	7	6	4
total	115	70	65	46

### Case study on breast, lung and colon cancers

We conduct experiments on three very important and common cancers, including Breast, Colon and Lung, to further study the ability of our proposed BRWLDA in predicting potential lncRNA-disease associations. Here, all known lncRNAs associated with the disease of interests are taken as seeds, and all candidate lncRNAs are ranked by BRWLDA according to their predicted probabilities (from high to low). For each type of cancer, we take the top 20 most plausible lncRNAs as candidates associated with this cancer. Next, we manually check these lncRNAs by mining biomedical literature from NCBI database (PMID IDs) and list the confirmed associations in Table 3.

**Table 3:** Breast, colon and lung cancers associated lncRNAs in the top 20 ranking lists of BRWLDA and the corresponding evidences

Cancer type	lncRNA	Evidence(PMID)	Rank
Breast	H19	16707459;14729626;12419837;22996375	1
Breast	HOTAIR	24721780;24531795;24829860	2
Breast	MALAT1	22492512;22996375;24499465	3
Breast	MEG3	14602737;	4
Breast	CDKN2B-AS1	17440112;20956613	5
Breast	PVT1	17908964;	7
Breast	NEAT1	2541770;23825647	11
Colon	HOTAIR	24667321;	1
Colon	MALAT1	22996375;	3
Colon	H19	21874233;22996375	4
Colon	MEG3	14602737	6
Colon	CCAT1	23416875	14
Lung	MALAT1	24499465;24757675;24817925	1
Lung	HOTAIR	24757675	2
Lung	H19	16707459;24499465;22996375	3
Lung	MEG3	14602737;	5
Lung	NEAT1	25818739;	10
Lung	BCYRN1	9422992;	14
Lung	TUG1	24853421;	15

Breast cancer is the second leading cause of female cancer death, comprises 22% of all cancers in women [57, 58]. Researchers state that some lncRNAs are strongly associated with the formation of breast cancer [59]. We apply BRWLDA to identify potential lncRNAs associated with breast cancer and seven of the top 20 ranks are verified by biomedical literature as the most potential candidates. Colon cancer is the third most common cancer worldwide and the most common human malignancies in western countries, its prevalence rate has been rapidly increasing [60]. Some critical mutations underlying the pathogenic mechanism of colon cancer are already confirmed. Especially mutations and dysregulations of some lncRNAs have close connection with the development of colon cancer [61]. BRWLDA correctly identified 5 colon cancer related lncRNAs. Lung cancer results in about 1.8 million deaths each year and its five years survival rate is approximately 15%, much lower than other cancer types [62, 63]. Recent data show that the risk of lung cancer mortality is even larger than the combination of the next three most common cancers (colon, breast and prostate) [64]. Recent researches have shown that lncRNAs play an important role in development and progression of lung cancer [64]. We make use of BRWLDA to identify the potential lung cancer-related lncRNAs, and seven lncRNAs are confirmed by biomedical literature. From these case studies, we can conclude that BRWLDA is powerful for predicting lncRNA-disease associations with a high level of reliability.

## MATERIALS AND METHODS

### LncRNA-disease associations

GeneRIFs [52] provides brief (up to 255 characters) functional descriptions of genes, miRNAs and lncRNAs in the NCBI database and contains gene specific information. The functional descriptions of these molecules could be annotated with controlled vocabularies, such as Disease Ontology [53] and Gene Ontology [45]. We downloaded the recent GeneRIF (access date: 2016-12-05) data and used an online tool named Open Biomedical Annotator [65, 66], which provides an ontology based web service to annotate public datasets with biomedical ontology concepts based on their textual metadata, to obtain the lncRNA-disease associations and lncRNA-GO terms associations. To avoid the limitation of single data source, we also collected lncRNA-disease associations from LncRNADisease database (access date: 2016-12-05). By integrating these two datasets, we obtained 837 lncRNA-disease associations between 240 lncRNAs and 232 diseases. If an lncRNA is associated with a disease term, this lncRNA is also associated with the ancestor diseases of that disease. By applying this expanding rule, we finally collected 2379 lncRNA-disease associations between 240 lncRNAs and 404 diseases for experiments.

### Disease semantic network constructed by disease ontology

We use Disease Ontology (DO) [53] hierarchy to construct the disease subnetwork of the bi-relational network, since DO is larger and provides greater coverage than MeSH (Medical Subject Headings) and OMIM (Online Mendelian Inheritance in Man) [67]. DO is a manually curated and disease-focused comprehensive subset of Unified Medical Language System. Similar to the Gene Ontology (GO) [45], DO organizes disease terms via a directed acyclic graph (DAG). Figure 5 shows an example of partial structure of DO, where each DOID represents a disease, direct edges encode the hierarchical relationship between diseases. Descendant DOIDs describe more specific diseases than their ancestor DOIDs. For example, ‘DO:1612’ (breast cancer) is a subtype of ‘DO:5093’ (thoracic cancer), which is the descendant of ‘DOID:0050686’ (organ system cancer). From Figure 5, we can find a new disease association of an lncRNA often corresponds to descendants of the diseases already associated with that lncRNA, because of the further rephrased relationship, accumulated biological data and knowledge. From this perspective, correctly identifying these new associations not only facilitates our understanding of disease mechanism in RNA level, but also boosts the pace of precise diagnosis and treatment.

Based on the DO hierarchical structure, we adopt a widely used method suggested by Wang et al. [68] to measure the semantic similarity between diseases. Suppose disease *d'* is an ancestor of disease *d* (or *d'* = *d*), the recursive definition of the contribution of *d'* to *d* is as follows:

$$S_{d}(d')=\{\begin{array}{c} 1,\\ \max(w_{e}\cdot S_{d}(d'')|d''\in \mathrm{children}\text{ of }d') \end{array}\;\begin{array}{c} d'=d\\ d'\neq d \end{array}$$ (1)

where *w_e_* is fixed as 0.9. The semantic similarity between two disease terms *d*_1_ and *d*_2_ is calculated as follows:

$$\mathrm{DS}(d_{1},d_{2})=\frac{\sum_{d'\in \mathrm{anc}(d_{1})\cap \mathrm{anc}(d_{2})}\left(S_{d_{1}}(d)+S_{d_{2}}(d)\right)}{\mathrm{SV}(d_{1})+\mathrm{SV}(d_{2})}$$ (2)

where *anc*(*d*_1_) includes the ancestors of disease *d*_1_ and itself, *SV*(*d*_1_) is the total semantic contribution of the term *d*_1_ and it is calculated as:

$$\mathrm{SV}(d_{1})=\sum_{d'\in \mathrm{anc}(d_{1})}S_{d_{1}}(d')$$ (3)

Suppose *G* ∈ ℝ^*D*×*D*^ (*D* is the number of diseases of interests) be the adjacency matrix of the DO DAG. If *d* is a direct child of *d'*, then *G*(*d'*, *d*) = 1, otherwise *G*(*d'*, *d*) = 0. As *DS* measures the hierarchical semantic similarity between any two diseases, to simulate random walk on the DAG, we initialize the transitional probability matrix *W*_*DD*_ ∈ ℝ^*D*×*D*^ on *G* as follows:

$$W_{DD}(d',d)=\mathrm{DS}(d',d)\times G(d',d)$$ (4)

From the above initialization, it is clear that a random walker can jump from *d'* to *d* in the first step only if *d'* and *d* have the parent-child relationship in the disease subnetwork. In this way, we can infer the more specific diseases of an lncRNA based on the diseases already associated with this lncRNA and hierarchical relationship between diseases.

### LncRNA similarity network constructed by multiple data sources

**Figure 6:**
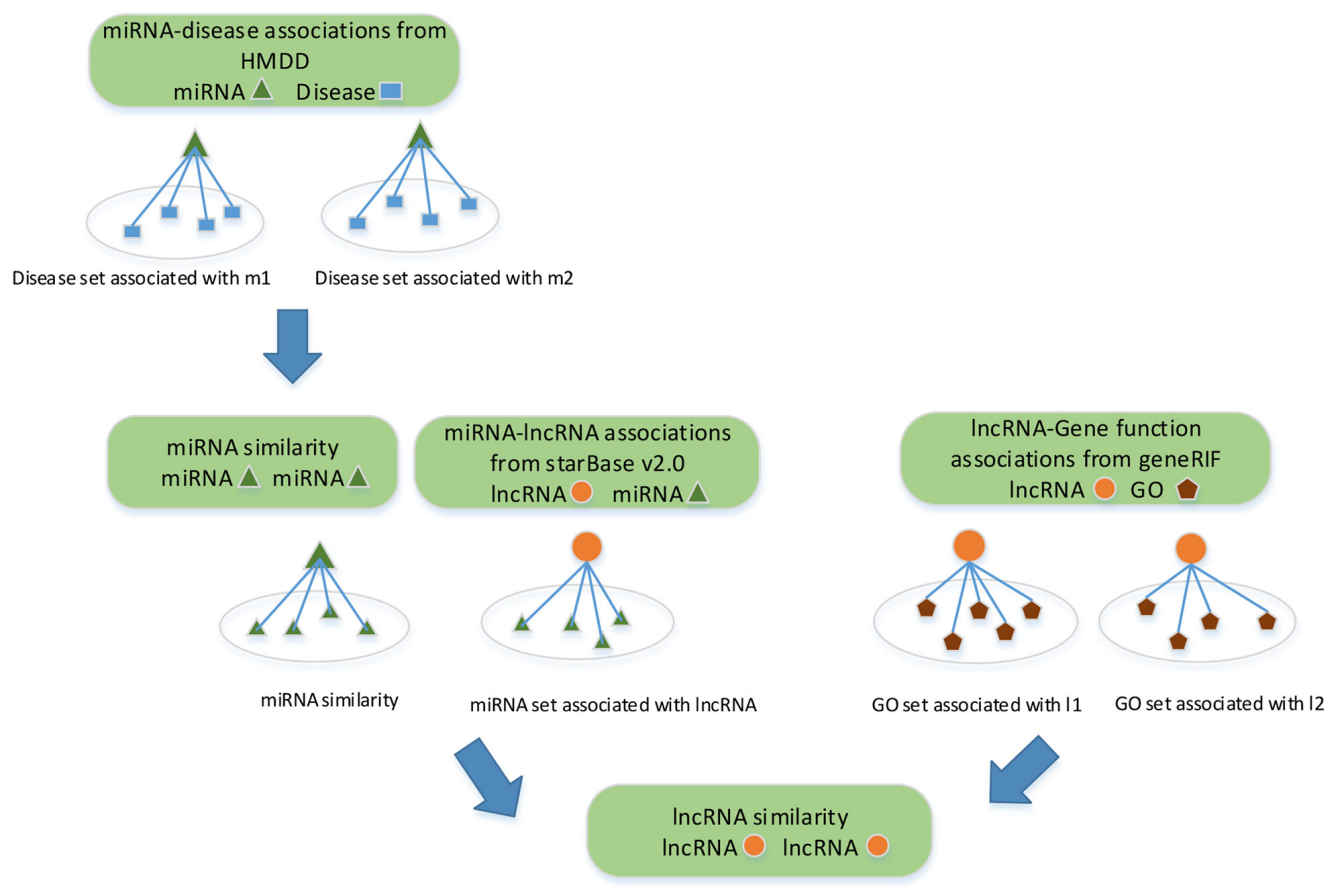
Flowchart of lncRNA similarity measurement Three different biological data related to lncRNAs are used to measure lncRNA-lncRNA similarity.

lncRNAs with similar functions are often associated with similar diseases. Many methods have been proposed to infer lncRNA-disease associations based on lncRNA similarity network [32, 43, 47–50]. These methods are either limited to single dataset, or biased the similarity since each testing association is already used to construct the lncRNA similarity network before the validation. Given that, we quantify the similarity between lncRNAs based on the collected lncRNA-miRNA interactions, miRNA-disease associations and lncRNA-gene function associations. Clearly, the similarity between lncRNAs is completely independent from lncRNA-disease associations. A flowchart of the lncRNA similarity measurement is shown in Figure 6.

The similarity between lncRNAs *l*_1_ and *l*_2_ is calculated as follows:

$$\mathrm{LS}(l_{1},l_{2})=\mathrm{FS}(l_{1},l_{2})+\mathrm{MDS}(l_{1},l_{2})$$ (5)

where *FS*(*l*_1_,*l*_2_) and *MDS*(*l*_1_,*l*_2_) represent the similarity derived from functional associations and miRNA-disease associations, respectively.

We use the Open Biomedical Annotator tool [65, 66] to parse geneRIFs and obtain 3406 associations between 240 lncRNAs and 582 GO terms. Since these associations are not strictly divided into three GO sub-ontologies and rather sparse compared with GO term space, it is inappropriate to use structure based algorithms to calculate the similarity between lncRNAs. Here, we utilize a Bayesian prior probability based method to simply measure the lncRNAs functional similarity. Let Tl1+ and Tl1l2+ represent the GO annotation set of lncRNA *l*_1_ and the common annotation set of *l*_1_ and *l*_2_, respectively. Then, the value of *FS*(*l*_1_, *l*_2_) is given as follows:

$$\mathrm{FS}(l_{1},l_{2})=\frac{\left|T_{l_{1}l_{2}}^{+}\right|}{\left|T_{l_{1}}^{+}\right|}$$ (6)

*MDS*(*l*_1_, *l*_2_) considers the similarity of lncRNA-associated miRNA groups. It has been proven that some lncRNAs act as competing endogenous RNAs in the regulation of gene expression [69]. The functional interactions between miRNAs and lncRNAs, and the crucial roles of miRNAs in various biological processes (including the affinity with genetic transcription and diseases) [70, 71] drive us to measure the lncRNA similarity based on lncRNA-miRNA associations. The similarity between two miRNAs (*m*_1_ and *m*_2_) is defined as follows:

$$\mathrm{MS}(m_{1},m_{2})=|D(m_{1})\cap D(m_{2})|$$ (7)

where *D*(*m*_1_) and *D*(*m*_2_) indicate the disease sets related to *m*_1_ and *m*_2_, respectively.

Suppose *M*(*l*_1_) and *M*(*l*_2_) are the known sets of miRNAs associated with lncRNAs *l*_1_ and *l*_2_, respectively, and *MDS*(*l*_1_, *l*_2_) can be computed as follows:

$$\mathrm{MDS}(l_{1},l_{2})=\frac{\sum_{m\in M(l_{1})}\overset{⌢}{M}S(m,M(l_{2}))+\sum_{m\in M(l_{2})}\overset{⌢}{M}S(m,M(l_{1}))}{\left|M(l_{1})|+|M(l_{2})\right|}$$ (8)

where $\overset{⌢}{M}S(m,M)=\max_{m_{i}\in M}\{\mathrm{MS}(m,m_{i})\}$. By sum-ming up *FS*(*l*_1_, *l*_2_) and *MDS*(*l*_1_, *l*_2_), we can get the similarity between lncRNAs. To ensure the similarity between pairwise lncRNAs between 0 and 1, we normalize *LS* as follows:

$$\overset{⌢}{L}S(l_{1},l_{2})=\frac{LS(l_{1},l_{2})}{\sqrt{D_{L}(l_{1},l_{1})\times D_{L}(l_{2},l_{2})}}$$ (9)

Where *D_L_* ∈ ℝ^*N*×*N*^ (*N* is the number of lncRNAs) is a diagonal matrix with $D_{L}(l_{i},l_{i})=\sum_{j=1}^{N}LS(l_{i},l_{j})$.

### Bi-Random walk with restart on bi-relational network

**Figure 7:**
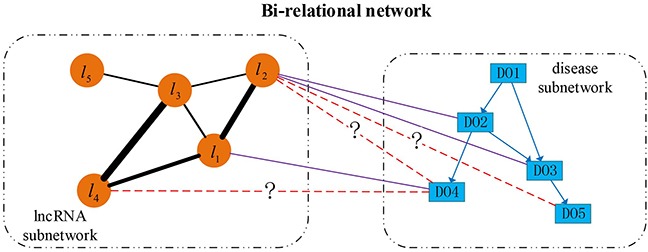
An illustrative example of directed bi-relational network Directed edges between disease nodes describe the hierarchical relationship between diseases, undirect edges between lncRNAs describe the functional similarity (reflected by line width) between lncRNAs, and undirect edges between diseases and lncRNAs indicate the known lncRNA-disease associations, and the dashed edges between lncRNAs and diseases indicate the novel associations.

Based on the directed disease subnetwork, lncRNA functional subnetwork and collected lncRNA-disease associations, we construct a directed bi-relational network, which includes two types of nodes (lncRNAs and DO terms) and three types of edges between them. An illustrative example of this bi-relational network is shown in Figure 7. In this figure, each rectangle indicates a DO term, each circular indicates an lncRNA. Direct edges represent the hierarchical relationship between diseases, undirected edges between lncRNAs denote the similarity between lncRNAs with the thickness representing the strength of similarity, undirected solid edges encode the known associations between diseases and lncRNAs, while dashed edges marked with question mark (?) represent the potential lncRNA-disease associations. lncRNAs (i.e., *l*_3_, *l*_4_ and *l*_5_) currently are not associated with any disease term but they can be associated with some disease terms. For example, there is a missing association between *l*_4_ and DO4. To avoid making Figure 7 too busy, we only show the direct associations between lncRNAs and diseases. In fact, ancestor diseases of directly associated diseases are also inherently associated with these lncRNAs. For example, *l*_1_ is not only associated with DO4, but also DO2 and DO1, since the latter two are ancestors of DO4.

From Figures 5 and 7, we find that newly associated diseases with an lncRNA often correspond to descendants of the diseases already associated with this lncRNA. For a disease already associated with some lncRNAs, we can identify other lncRNAs also associated this disease based on the functional relationship between lncRNAs. From Figure 7, we can see that the lncRNA subnetwork is an undirect one and the disease subnetwork is a directed one. To account for the structural difference between these two subnetworks, and to identify new diseases (or lncRNAs) associated with lncRNAs (or diseases), we perform bi-random walks with restart on the directed bi-relational network illustrated in Figure 7.

Let *A_LD_* ∈ ℝ^*N*×*D*^ store the known lncRNA-disease associations between *N* lncRNAs and *D* diseases. If an lncRNA is associated with disease *d* or its descendants, *A_LD_*(*l*,*d*) = 1; otherwise *A_LD_*(*l*,*d*) = 0. Suppose ‘DO4’ should be associated with *l*_2_ and its parental ‘DO2’ is already associated with the same lncRNA, we can take *l*_2_ as the starting node for a random walker, which jumps to ‘DO2’ and then downward to ‘DO4’ along the edges of bi-relational network. As a result, we can get the probabilistic association between *l*_2_ and ‘DO4’, and thus to estimate the credibility of this association. To realize this process, we apply random walks with restart by taking diseases already associated with an lncRNA as intermediate nodes to predict new diseases associated with the same lncRNA as follows:

$$F_{D}^{t}(l,d)=\{\begin{array}{cc} \begin{array}{l} \alpha \sum_{d'\in \mathrm{par}(d)}F_{D}^{t-1}(l,d')W_{DD}(d',d)\\ +(1-\alpha )A_{LD}(l,d) \end{array} & t\leq t_{d}\\ F_{D}^{t-1}(l,d) & t>t_{d} \end{array}$$ (10)

Where *par*(*d*) includes the direct parent diseases of *d*, *W_DD_* ∈ ℝ^*D*×*D*^ stores the transitional probabilities between *D* diseases and it is initialized by Eq. (4), $F_{D}^{t}(l,d)$ is the predicted relevance between lncRNA *l* and disease *d* in the *t*-th iteration, FD0=ALD, *α* is a parameter to control the restart probability for a random walker, and *t_d_* is the specified maximal steps for a random walker jumping in the disease subnetwork. If *t* > *t_d_*, the walker will not jump in the subnetwork consisted with diseases, and $F_{D}^{t}=F_{D}^{t_{d}}$. In fact, the above equation is also motivated by the observations that semantically related diseases tend to be associated with the same lncRNAs [20]. We want to remark that although a disease's associated lncRNAs are completely unknown, the above equation can still identify some lncRNAs, which are probably associated with this disease.

A random walker can also jump from DO4 to *l*_1_ and then to *l*_4_. In this way, we can infer diseases associated with *l*_4_, even if the associated diseases of *l*_4_ are temporarily completely unknown. To mimic this process, we apply random walks with restart on the lncRNA subnetwork as follows:

$$F_{L}^{t}(l,d)=\{\begin{array}{cc} \begin{array}{l} \alpha \sum_{l'=1}^{N}\overset{⌢}{L}S(l,l')F_{L}^{t-1}(l',d)\\ +(1-\alpha )A_{LD}(l,d) \end{array} & t\leq t_{l}\\ F_{L}^{t-1}(l,d) & t>t_{l} \end{array}$$ (11)

Where *t_l_* is the specified maximal step for a random walker jumping in the lncRNA subnetwork, $F_{L}^{t}(l,d)$ is the predicted relevance between lncRNA *l* and disease *d* in the *t*-th iteration, $F_{L}^{0}=A_{LD}$. Similarly, the random walker will stop jumping when *t* > *t_l_*, and $F_{L}^{t}=F_{L}^{t_{l}}$.

After the bi-random walks in the disease subnetwork and in the lncRNA subnetwork in the *t*-th step, BRWLDA further combines $F_{L}^{t}$ and $F_{D}^{t}$ into *F^t^* as follows:

$$F_{D}^{t}=F_{L}^{t}=F^{t}=\frac{F_{D}^{t}+F_{L}^{t}}{2}$$ (12)

By iteratively applying Eq. (9), Eq. (10) and Eq. (11), we can obtain the potential lncRNA-disease associations among *N* lncRNAs and *D* diseases. Obviously, the larger the value of *F^t^*(*l*, *d*), the more probable lncRNA *l* associated with disease *d* is.

## DISCUSSION

Increasing evidences show that lncRNAs play essential roles in various biological processes and they have association with various complex human diseases [7–12]. Researchers have been attempting to identify lncRNAs associated with diseases by biological experiments and some identified lncRNAs are already used as biomarkers for clinical diagnosis, prognosis and treatment [13–17]. However, the wet-lab experiment based identification is too expensive, time consuming, and low throughput. These identified lncRNAs and accumulated various biological data enable to develop computational models to predict additional lncRNA-disease associations in large scale, which could boost the pace of follow-up wet-lab experimental verification and save resources. We propose a computational model called BRWLDA to predict lncRNA-disease associations by bi-random walks on a directed bi-relational network. The bi-relational network is built up with two subnetworks, namely lncRNA-lncRNA functional similarity network derived from various biological data, and disease-disease similarity network derived from the ontological structure between diseases, and inter-connections between these two subnetworks setup by available lncRNA-disease associations. Compared with five state-of-the-art related computational models [22, 23, 32, 49, 48], BRWLDA achieves the highest AUCs of 0.7952 and 0.7940 in the lncRNA and disease oriented LOOCV. In the recursively masked experiment, a more challenging and rarely studied experimental protocol, BRWLDA again obtains the highest AUCs of 0.9888, 0.9548, 0.9275, with 1, 3, 5 diseases masked for an lncRNA. Case study on 6 critical cancers (including Breast, Colon, Bladder, Liver, Lung and Stomach), whose related lncRNAs are completely masked (or unknown), shows that BRWLDA also obtains more related lncRNAs than other comparing methods. In addition, manually literature mining also confirms that, many of the top-20 plausible lncRNAs predicted by BRWLDA are indeed associated with Breast, Colon and Lung cancers. These comparative experiments suggest that BRWLDA would greatly boost the identification of lncRNA-disease associations.

Several components contribute to the high predictive performance of BRWLDA. First, multiple biological datasets, including lncRNA-miRNA associations, miRNA-disease associations and lncRNA-gene function associations, are utilized to establish a more functional coherent lncRNA-lncRNA network. Second, BRWLDA not only employs the knowledge that functional similar lncRNAs are associated with semantic similar diseases, and vice versa, but also uses the pattern that newly discovered diseases associated with an lncRNA usually correspond to descendants of the diseases that are already associated with this lncRNA. BRWLDA employs this pattern by directed random walks with restart on the disease hierarchical network. In practice, some comparing methods (i.e., RWRHLD, IRWRLDA) also take advantage of the same semantic measure between diseases and a similar heterogeneous network as the directed bi-relational network adopted by BRWLDA. But they apply global random walk with restart on the whole network. They neither concretely employ the ontological structure in the prediction process, nor the pattern of newly discovered diseases associated with lncRNAs. Third, BRWLDA can make predictions for new diseases (lncRNAs) whose associated lncRNAs (diseases) are completely unknown, since it does not solely depend on available lncRNA-disease associations. This feature greatly improves the practicability and reliability of BRWLDA. These predictions can be either made via a random walker jumping from lncRNA-lncRNA subnetwork to disease-disease hierarchical subnetwork, and vice versa. Fourth, BRWLDA provides options for user to prioritize diseases associated with lncRNAs, or prioritize lncRNAs associated with diseases, and the prioritization can be separately controlled by the maximum steps of bi-random walk. Last but not least, BRWLDA can easily work together with more biological information (i.e., lncRNA or miRNA-related interactions, disease phenotypic profiles and gene-disease associations) to identify candidate lncRNAs (or diseases) for all diseases (or lncRNAs) of interests, and its performance can be further improved by fusing these biological information.

There are several avenues to improve the performance of BRWLDA. For examples, there are still many other important data could be utilized, such as amino acid sequences and transcription information [72]. The collected lncRNA-miRNA associations, miRNA-disease associations and lncRNA-gene function associations are sparse and far from complete; they may also include some noisy associations. Incomplete and noisy associations heavily impact the performance of BRWLDA. Although we integrate heterogeneous data to remedy this issue, the performance of BRWLDA can be improved with more reliable associations available. The transitional probability between diseases is only empirically estimated, more accurate estimation and the mature of DO hierarchy can also enhance the ability of BRWLDA in identifying new lncRNAs associated with diseases. Complex diseases are not only related to lncRNAs, but also related to other biological molecules (i.e., miRNAs, genes and proteins), modelling all these related molecules in a complex network could vividly unveil the mechanism of complex diseases.

## References

[1] Crick F, Barnett L, Brenner S, Watts-Tobin R (1961). General nature of the genetic code for proteins. Nature.

[2] Bertone P, Stolc V, Royce TE, Rozowsky JS, Urban AE, Zhu X, Rinn JL, Tongprasit W, Samanta M, Weissman S, Gerstein M, Snyder M (2004). Global identification of human transcribed sequences with genome tiling arrays. Science.

[3] Birney E, Stamatoyannopoulos JA, Dutta A, Guigo R, Gingeras TR, Margulies EH, Weng Z, Snyder M, Dermitzakis ET, Thurman RE, Kuehn MS, Taylor CM, ENCODE Project Consortium (2007). Identification and analysis of functional elements in 1% of the human genome by the ENCODE pilot project. Nature.

[4] Core LJ, Waterfall JJ, Lis JT (2008). Nascent RNA sequencing reveals widespread pausing and divergent initiation at humanpromoters. Science.

[5] Kapranov P, Cheng J, Dike S, Nix DA, Duttagupta R, Willingham AT, Stadler PF, Herte J, Hackermüller J, Hofacker IL, Bell I, Cheung E, Gingeras TR (2007). RNA maps reveal new RNA classes and a possible function for pervasive transcription. Science.

[6] Kapranov P, Willingham AT, Gingeras TR (2007). Genome-wide transcription and the implications for genomic organization. Nat Rev Genet.

[7] Wang KC, Chang HY (2011). Molecular mechanisms of long noncoding RNAs. Mol Cell.

[8] Esteller M (2011). Non-coding RNAs in human disease. Nat Rev Genet.

[9] Wapinski O, Chang HY (2011). Long noncoding RNAs and human disease. Trends Cell Biol.

[10] Bai W, Yang W, Wang W, Wang Y, Liu C, Jiang Q, Hua J, Liao M (2017). GED: a manually curated comprehensive resource for epigenetic modification of gametogenesis. Brief Bioinform.

[11] Zhang Z, Zhang J, Fan C, Tang Y, Deng L (2017). KATZLGO: large-scale prediction of LncRNA functions by using the KATZ measure based on multiple networks. IEEE/ACM Trans Comput Biol Bioinform.

[12] Xiao Y, Zhang Z, Deng L (2017). Prediction of lncRNA-protein interactions using HeteSim scores based on heterogeneous networks. Sci Rep.

[13] Tsai MC, Manor O, Wan Y, Mosammaparast N, Wang JK, Lan F, Shi Y, Segal E, Chang HY (2010). Long noncoding RNA as modular scaffold of histone modification complexes. Science.

[14] Gupta RA, Shah N, Wang KC, Kim J, Horlings HM, Wong DJ, Tsai MC, Hung T, Argani P, Rinn JL, Wang YL, Brzoska P, Kong B (2010). Long non-coding RNA HOTAIR reprograms chromatin state to promote cancer metastasis. Nature.

[15] Brannan CI, Dees EC, Ingram RS, Tilghman SM (1990). The product of the H19 gene may function as an RNA. Mol Cell Biol.

[16] Barsyte-Lovejoy D, Lau SK, Boutros PC, Khosravi F, Jurisica I, Andrulis IL, Tsao MS, Penn LZ (2006). The c-Myc oncogene directly induces the H19 noncoding RNA by allele-specific binding to potentiate tumorigenesis. Cancer Res.

[17] Ariel I, Sughayer M, Fellig Y, Pizov G, Ayesh S, Podeh D, Libdeh BA, Levy C, Birman T, Tykocinski ML, de Groot N, Hochberg A (2000). The imprinted H19 gene is a marker of early recurrence in human bladder carcinoma. Mol Pathol.

[18] Chen G, Wang Z, Wang D, Qiu C, Liu M, Chen X, Zhang Q, Yan G, Cui Q (2013). LncRNADisease: a database for long-non-coding RNA-associated diseases. Nucleic Acids Res.

[19] Ning S, Zhang J, Wang P, Zhi H, Wang J, Liu Y, Gao Y, Guo M, Yue M, Wang L, Li X (2016). Lnc2Cancer: a manually curated database of experimentally supported lncRNAs associated with various human cancers. Nucleic Acids Res.

[20] Chen X, Yan C, Zhang X, You Z (2016). Long non-coding RNAs and complex diseases: from experimental results to computational models. Brief Bioinform.

[21] Jalali S, Kapoor S, Sivadas A, Bhartiya D, Scaria V (2015). Computational approaches towards understanding human long non-coding RNA biology. Bioinformatics.

[22] Chen X, Yan GY (2013). Novel human lncRNA-disease association inference based on lncRNA expression profiles. Bioinformatics.

[23] Sun J, Shi H, Wang Z, Zhang C, Liu L, Wang L, He W, Hao D, Liu S, Zhou M (2014). Inferring novel lncRNA-disease associations based on a random walk model of a lncRNA functional similarity network. Mol Biosyst.

[24] Tong H, Faloutsos C, Pan JY (2008). Random walk with restart: fast solutions and applications. Knowl Inf Syst.

[25] Yang X, Gao L, Guo X, Shi X, Wu H, Song F, Wang B (2014). A network based method for analysis of lncRNA-disease associations and prediction of lncRNAs implicated in diseases. PLoS One.

[26] Li JH, Liu S, Zhou H, Qu L, Yang J (2014). starBase v2.0: decoding miRNA-ceRNA, miRNA-ncRNA and protein-RNA interaction networks from large-scale CLIP-Seq data. Nucleic Acids Res.

[27] Amaral PP, Clark MB, Gascoigne DK, Dinger ME, Mattick JS (2011). lncRNAdb: a reference database for long noncoding RNAs. Nucleic Acids Res.

[28] Dinger ME, Pang KC, Mercer TR, Crowe ML, Grimmond SM, Mattick JS (2009). NRED: a database of long noncoding RNA expression. Nucleic Acids Res.

[29] Bu D, Yu K, Sun S, Xie C, Sk032ogerbø G, Miao R, Xiao H, Liao Q, Luo H, Zhao G, Zhao H, Liu Z, Liu C, Chen R (2012). NONCODE v3.0: integrative annotation of long noncoding RNAs. Nucleic Acids Res.

[30] Liu MX, Chen X, Chen G, Cui Q, Yan G (2014). A computational framework to infer human disease-associated long noncoding RNAs. PLoS One.

[31] Li J, Gao C, Wang Y, Ma W, Tu J, Wang J, Chen Z, Kong W, Cui Q (2014). A bioinformatics method for predicting long noncoding RNAs associated with vascular disease. Sci China Life Sci.

[32] Zhou M, Wang X, Li J, Hao D, Wang Z, Shi H, Han L, Zhou H, Sun J (2015). Prioritizing candidate disease-related long non-coding RNAs by walking on the heterogeneous lncRNA and disease network. Mol Biosyst.

[33] Chen X (2015). Predicting lncRNA-disease associations and constructing lncRNA functional similarity network based on the information of miRNA. Sci Rep.

[34] Chen X, Yan CC, Zhang X, You ZH, Deng L, Liu Y, Zhang Y, Dai Q (2016). WBSMDA: within and between score for MiRNA-disease association prediction. Sci Rep.

[35] Chen X, Liu MX, Yan GY (2012). RWRMDA: predicting novel human microRNA–disease associations. Mol Biosyst.

[36] You ZH, Huang ZA, Zhu Z, Yan G, Li ZW, Wen Z, Chen X (2017). PBMDA: A novel and effective path-based computational model for miRNA-disease association prediction. PLoS Comput Biol.

[37] Zeng X, Zhang X, Zou Q (2016). Integrative approaches for predicting microRNA function and prioritizing disease-related microRNA using biological interaction networks. Brief Bioinform.

[38] Liu Y, Zeng X, He Z, Zou Q (2016). Inferring microRNA-disease associations by random walk on a heterogeneous network with multiple data sources. IEEE/ACM Trans Comput Biol Bioinform.

[39] Tang W, Liao Z, Zou Q (2016). Which statistical significance test best detects oncomiRNAs in cancer tissues? An exploratory analysis. Oncotarget.

[40] Zou Q, Li J, Hong Q, Lin Z, Shi H, Wu Y, Ju Y (2015). Prediction of microRNA-disease associations based on social network analysis methods. Biomed Res Int.

[41] Yu G, Fu G, Wang J, Zhu H (2015). Predicting protein function via semantic integration of multiple networks. IEEE/ACM Trans Comput Biol Bioinform.

[42] Li H, Menon R, Omenn G, Guan Y (2014). The emerging era of genomic data integration for analyzing splice isoform function. Trends Genet.

[43] Chen X (2015). KATZLDA: KATZ measure for the lncRNA-disease association prediction. Sci Rep.

[44] Lan W, Li M, Zhao K, Liu J, Wu FX, Pan Y, Wang J (2016). LDAP: a web server for lncRNA-disease association prediction. Bioinformatics.

[45] Ashburner M, Ball CA, Blake JA, Botstein D, Butler H, Cherry JM, Davis AP, Dolinski K, Dwight SS, Eppig JT, Harris MA, Hill DP, Issel-Tarver L (2000). Gene ontology: tool for the unification of biology. The Gene Ontology Consortium. Nat Genet.

[46] Zakeri P, Jeuris B, Vandebril R, Moreau Y (2014). Protein fold recognition using geometric kernel data fusion. Bioinformatics.

[47] Chen X, You Z, Yan G, Gong D (2016). IRWRLDA: improved random walk with restart for lncRNA-disease association prediction. Oncotarget.

[48] Zhang J, Zhang Z, Chen Z, Deng L (2017). Integrating multiple heterogeneous networks for novel LncRNA-disease association inference. IEEE/ACM Trans Comput Biol Bioinform.

[49] Huang YA, Chen X, You ZH, Huang D, Chan KC (2016). ILNCSIM: improved lncRNA functional similarity calculation model. Oncotarget.

[50] Chen X, Huang YA, Wang XS, You ZH, Chan KC (2016). FMLNCSIM: fuzzy measure-based lncRNA functional similarity calculation model. Oncotarget.

[51] Li Y, Qiu C, Tu J, Geng B, Yang J, Jiang T, Cui Q (2014). HMDD v2.0: a database for experimentally supported human microRNA and disease associations. Nucleic Acids Res.

[52] Lu Z, Cohen KB, Hunter L (2007). GeneRIF quality assurance as summary revision. Pac Symp Biocomput.

[53] Schriml LM, Arze C, Nadendla S, Chang YW, Mazaitis M, Felix V, Feng G, Kibbe WA (2012). Disease ontology: a backbone for disease semantic integration. Nucleic Acids Res.

[54] Xie M, Hwang T, Kuang R (2012). Prioritizing disease genes by bi-random walk. Pacific-Asia Conference on Advances in Knowledge Discovery and Data Mining. Springer-Verlag.

[55] Song JY, Lee JH, Joe CO, Lim D, Chung J (2011). Retrotransposon-specific DNA hypomethylation and two-step loss-of-imprinting during WW45 haploinsufficiency-induced hepatocarcinogenesis. Biochem Biophys Res Commun.

[56] Zhang E, Han L, Yin D, Kong R, De W Chen J (2014). c-Myc-induced, long, noncoding H19 affects cell proliferation and predicts a poor prognosis in patients with gastric cancer. Med Oncol.

[57] Donahue HJ, Genetos DC (2013). Genomic approaches in breast cancer research. Brief Funct Genomics.

[58] Karagoz K, Sinha R, Arga KY (2015). Triple negative breast cancer: a multi-omics network discovery strategy for candidate targets and driving pathways. OMICS.

[59] Meng J, Li P, Zhang Q, Yang Z, Fu S (2014). A four-long non-coding RNA signature in predicting breast cancer survival. J Exp Clin Cancer Res.

[60] Xiang JF, Yin QF, Chen T, Zhang Y, Zhang X, Wu Z, Zhang S, Wang H, Ge J, Lu X, Yang L, Chen L (2014). Human colorectal cancer-specific CCAT1-L lncRNA regulates long-range chromatin interactions at the MYC locus. Cell Res.

[61] Wood LD, Parsons DW, Jones S, Lin J, Sjöblom T, Leary RJ, Shen D, Boca SM, Barber T, Ptak J, Silliman N, Szabo S, Dezso Z (2007). The genomic landscapes of human breast and colorectal cancers. Science.

[62] Gutschner T, Hämmerle M, Eißmann M, Hsu J, Kim Y, Hung G, Revenko A, Arun G, Stentrup M, Groß M, Zörnig M, MacLeod AR, Spector DL (2013). The noncoding RNA MALAT1 is a critical regulator of the metastasis phenotype of lung cancer cells. Cancer Res.

[63] Liu X, Liu Z, Sun M, Liu J, Wang Z, De W (2013). The long non-coding RNA HOTAIR indicates a poor prognosis and promotes metastasis in non-small cell lung cancer. BMC Cancer.

[64] White NM, Cabanski CR, Silva-Fisher JM, Dang HX, Govindan R, Maher CA (2014). Transcriptome sequencing reveals altered long intergenic non-coding RNAs in lung cancer. Genome Biol.

[65] Jonquet C, Shah N, Musen M (2009). The open biomedical annotator. Summit Translat Bioinforma.

[66] Hu Y, Zhou W, Ren J, Dong L, Wang Y, Jin S, Cheng L (2016). Annotating the function of the human genome with gene ontology and disease ontology. Biomed Res Int.

[67] Hamosh A, Scott AF, Amberger JS, Bocchini CA, McKusick VA (2005). Online Mendelian Inheritance in Man (OMIM), a knowledgebase of human genes and genetic disorders. Nucleic Acids Res.

[68] Wang D, Wang J, Lu M, Song F, Cui Q (2010). Inferring the human microRNA functional similarity and functional network based on microRNA-associated diseases. Bioinformatics.

[69] Xia T, Liao Q, Jiang X, Shao Y, Xiao B, Xi Y, Guo J (2014). Long noncoding RNA associated-competing endogenous RNAs in gastric cancer. Sci Rep.

[70] Xu Y, Guo M, Liu X, Wang C, Liu Y (2014). Inferring the soybean (Glycine max) microRNA functional network based on target gene network. Bioinformatics.

[71] Xu Y, Guo M, Liu X, Wang C, Liu Y, Liu G (2016). Identify bilayer modules via pseudo-3D clustering: applications to miRNA-gene bilayer networks. Nucleic Acids Res.

[72] Li L, Chen Z, Zhang L, Liu G, Hua J, Jia L, Liao M (2016). Genome-wide targets identification of “core” pluripotency transcription factors with integrated features in human embryonic stem cells. Mol Biosyst.

